# Economic Policy Uncertainty (EPU) and China's Export Fluctuation in the Post-pandemic Era: An Empirical Analysis based on the TVP-SV-VAR Model

**DOI:** 10.3389/fpubh.2021.788171

**Published:** 2021-12-06

**Authors:** Guoheng Hu, Shan Liu

**Affiliations:** School of Business, Henan Normal University, Xinxiang, China

**Keywords:** post-pandemic era, economic policy uncertainty (EPU), China's exports, dynamic relations, TVP-SV-VAR model

## Abstract

In the COVID-19 pandemic, the bidirectional policy adopted by the governments to stimulate domestic economy and reinforce foreign trade control is making the trade environment abnormally complex. China is facing a new challenge in export trade growth. Based on the continuous monthly data from January 2002 to April 2021, this paper uses the time-varying TVP-SV-VAR model to study the impulse response of China's export trade to economic policy uncertainty (EPU). It is found that (1) on the whole, the shock of global EPU and China's EPU on China's export to the OBOR/RCEP member countries is time-varying, different, and structurally significant; (2) during the pandemic, EPU has a significant short-term negative shock on China's gross exports and export to OBOR/RCEP members, and this shock is especially big in the case of global EPU. In the post-pandemic era, China should strengthen pandemic control and economic risk monitoring, continue with execution of multilateral FTAs and create a sustainably stable export trade environment.

## Introduction

**Figure 1:**
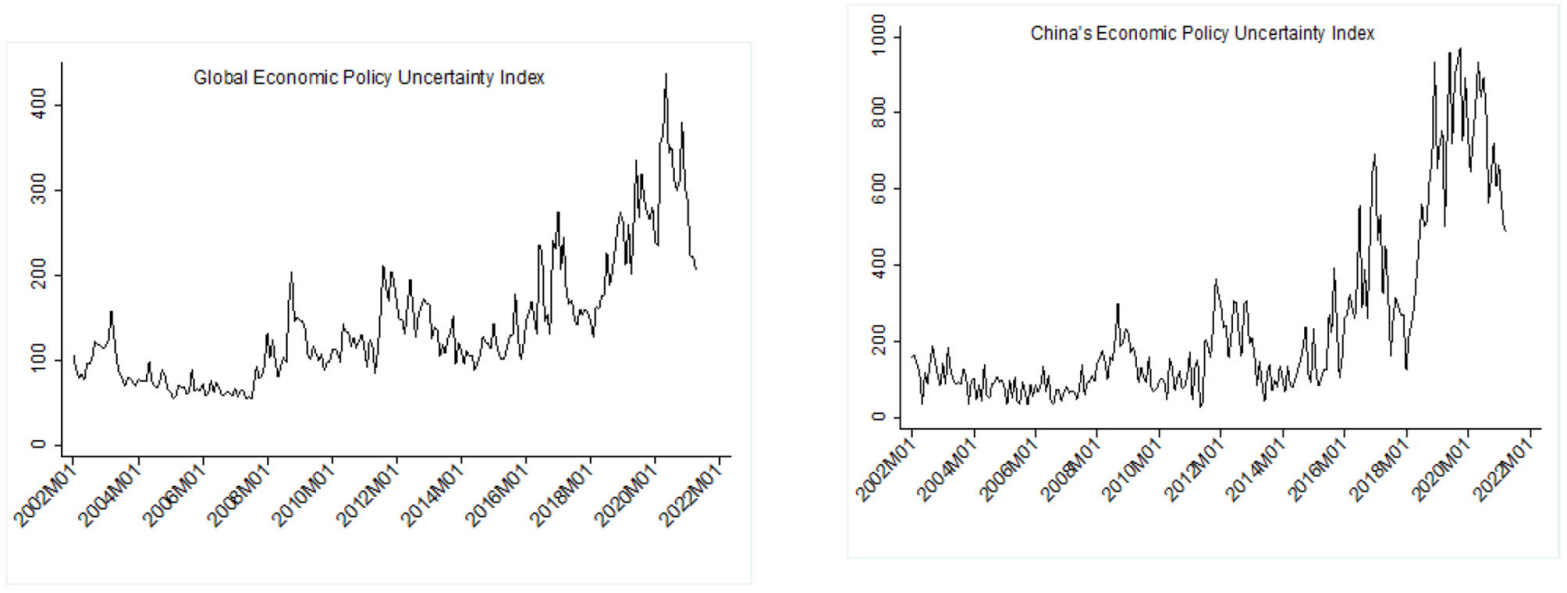
EPU index trend chart: Global vs. China.

The outbreak of the COVID-19 pandemic has brought intense shocks on normal economic activities and economic exchange among nations. To moderate the shock, upon the pandemic breaking out the governments released stimulation policies which resulted in an upsurge of economic policy uncertainty (EPU). If we observe the global EPU index and China's EPU index (shown in Figure 1), we can identify an obvious upsurge in the trend of both, with the peaks corresponding respectively to the SARS epidemic (2003), the financial crisis (2008), the China-U.S. trade war (2018), and the COVID-19 pandemic (2020). China has an EPU index greater than the globe, because the 2018 China-U.S. trade friction worsened this EPU. The subsequent pandemic drove the EPU index to a new high which was characterized by extremely obvious fluctuations at the height of the pandemic. As China's preventive and control measures achieved a stable effect, its EPU dropped remarkably with the dawn of the post-pandemic era, with these measures becoming the normal.

**Figure 2:**
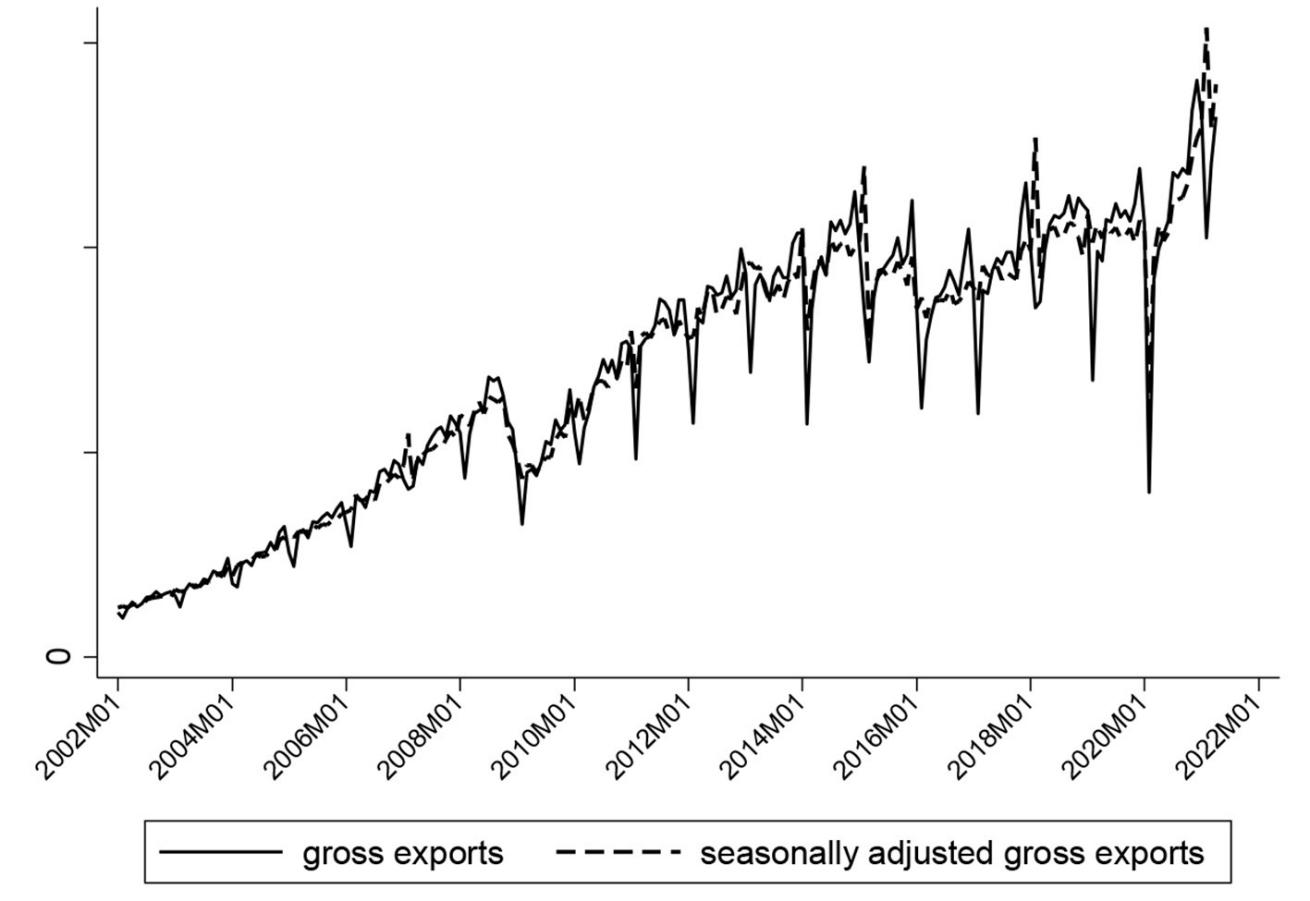
China's gross exports trend chart.

As opposed to the 2008 financial crisis when the governments responded with unidirectional domestic and foreign policies, the COVID-19 pandemic forced the governments to implement a fiscal stimulus approach to pandemic alleviation at home as well as stringent foreign trade control, e.g., trade protection, to get the contagion under control as part of foreign policy. Unquestionably the post-pandemic era is confronting China with a much more complex context of policy. Figure 2 provides the overall change in China's gross exports. On the whole, since the financial crisis, gross exports have slowed up in growth with a remarkably widened range of fluctuations, let alone the pandemic whose short-term shock on export trade hit the extrema. Thanks to the governments' effective control, China's export trade rebounded rapidly following a short-lived extreme shock, promising largely long-term stability.

In the post-pandemic era, bidirectional policy adopted by the governments to stimulate domestic economy and reinforce foreign trade control is making the trade environment abnormally complex. Besides, possible repeal of the stimulus policy combined with the Delta mutant-caused local recurrence may pose a new challenge to China's export growth. In order to explore the shock mechanism of internal and external uncertainties concerning China's export trade as well as how it evolves dynamically, this paper took the pandemic as an important event. A time-varying parameter stochastic volatility vector autoregression model (TVP-SV-VAR model) is developed to include the global EPU index, China's EPU index, global gross exports, China's gross exports, global gross outputs, China's gross outputs, and the continuous monthly history of renminbi (RMB) exchange rates. By setting up equal intervals and the time point-based impulse response function, this paper analyzes at length the shock of China's EPU on China's gross exports, including exports to OBOR/RCEP member countries, as well as the relevant differentials. Based on empirical research, this paper further investigates the practicable approaches China should take in response to the internal and external EPU risks in the post-pandemic era with the aim of making export trade grow steadily.

The paper has three contribution margins. First, the time-varying parameter vector autoregression (TVP-VA) model is constructed in place of the traditional vector autoregression (VAR) model so that highly time-effective monthly data from January 2002 to April 2021 help observe EPU's dynamic shock on China's export trade. Second, the pandemic is chosen as an important event and the continuous month-based history of RMB exchange rate changes and the financial crisis as contrast events so as to dynamically clarify the shock of post-pandemic EPU on China's export trade. Third, a study is made to investigate the heterogeneity of the shock of the internal and external EPUs on export trade between China and the OBOR/RCEP members.

## Literature Review

EPU is an important component of economic uncertainty. Specifically, it means the failure of the government to clarify the direction and intensity of economic policy anticipation, and why implementation and stance change cause economic entities to fail to precisely predict whether, when, and how to change uncertainties brought by existing economic policy (1, 2). EPU has an obvious effect on the main macroeconomic variables. A short-term EPU rise has a negative shock on China's overall economic growth, investment, and consumption (3), with the latter two reacting most saliently (4). EPU disturbs the smooth flow of international capital (5) and there is an nonlinear influence on normal capital flow in emerging economies (6). EPU decreases the employment rate by a large margin and there is a big differential in this inhibitive effect between differently-owned firms (7). The negative effect is thought to be closely correlated with a country's economic development level (8). More research indicates that in the context of EPU, the behavioral anomaly of market entities is the microfoundation of negative shocks. EPU produces an inhibitive effect on a firm's investment behavior through cash cost and marginal income (9). EPU, such as trade protection policy uncertainty, has an inhibitive effect on the firm's investment behavior (10). A time-varying parameter factor-augmented auto regression (TVP-FAVAR) model-based study found that EPU increases often come with overall bank credit falls and this effect is obviously time-varying (11).

EPU's shock on export trade is represented, first of all, in terms of trade volume and product quality (12). Rises in an export country's EPU drive up the export intention and gross exports of a domestic firm, while for the EPU of the country of destination, there is an inhibitive effect (13). EPU of the country of destination not only inhibits China's gross exports (14) but also affects export product quality (15). Increases in a country's EPU have a remarkable inhibitive effect on the price and quality of its export goods (16). An empirical study introduced the augmented gravity model, proving EPU constrains the intensive margin and extensive margin of China's export trade (17). It was further proved that this uncertainty will have repercussions on global trade (18). Some scholars suggested that EPU increases result in remarkable decreases in high-tech product exports while impacting positively on price margin (19).

EPU impacts a country's export trade mainly through the change in export demand and import demand. When global EPU climbs, the governments concentrate more on domestic economy than on import demand in consideration of rational expectations in order to smooth the adverse shock of global EPU (20). When China's EPU rises, the financial ripple effect will aggravate corporate financing constraints, so the upstream firm may find it difficult to meet normal export demand (21). In the post-pandemic era, the discretionary approach taken by the governments to mitigating the various hazards that might arise at any time adds to EPU to a certain degree, whereas if a member country joins a regional trade organization or signs a preferential trade agreement, EPU will fall, thereby lowering the pressure on export trade on both the supply and the demand sides (22).

The above investigations differentiate internal uncertainty from external uncertainty and reveal the effect on China's export trade and the relevant mechanism. However, most are based on traditional annual data, so the findings represent the population mean, rather than the time-varying characteristics of the shock. In fact, both EPU and gross exports are time-varying concerning the difference between the short-term fluctuation and the long-term trend. For this reason, this paper uses the time-varying TVP-SV-VAR model and time-effective monthly data for the complex, volatile post-pandemic context of trade. This is intended to more precisely unveil how the shock of external EPU and internal EPU on China's export trade evolves dynamically. As a result, a scientific decision-making basis is provided for stable export trade.

## Model Specification and Variable Selection

In time series vector auto regression (VAR), the assumption of homoscedasticity leads to the result being only statistically significant and the time-varying characteristics cannot be reflected. The time-varying parameter stochastic volatility vector auto regression (TVP-SV-VAR) model cancels this assumption while assuming that the time-varying parameters are stochastically variable. If we take samples using the Markov chain Monte Carlo method (MCMC) in Bayesian inference, we can correctly identify dynamic evolution characteristics of the economic variables. In order to analyze the shock of China's export trade to EPU during the pandemic, a TVP-SV-VAR model is specified as follows:


(1) $$\begin{array}{r} y_{t}=X_{t}\beta +A^{-1}\sum_{t}\varepsilon_{t},t=s+1,\cdots \;,n \end{array}$$

Where, parameters β, A, and Σ are assumed to follow a first-order random walk and are time-varying; and *y*_*t*_ are the *K*× one-dimensional vectors with four k-dimensional endogenous variables, including China's gross exports, EPU, gross outputs, and the RMB exchange rate.

**Figure 3:**
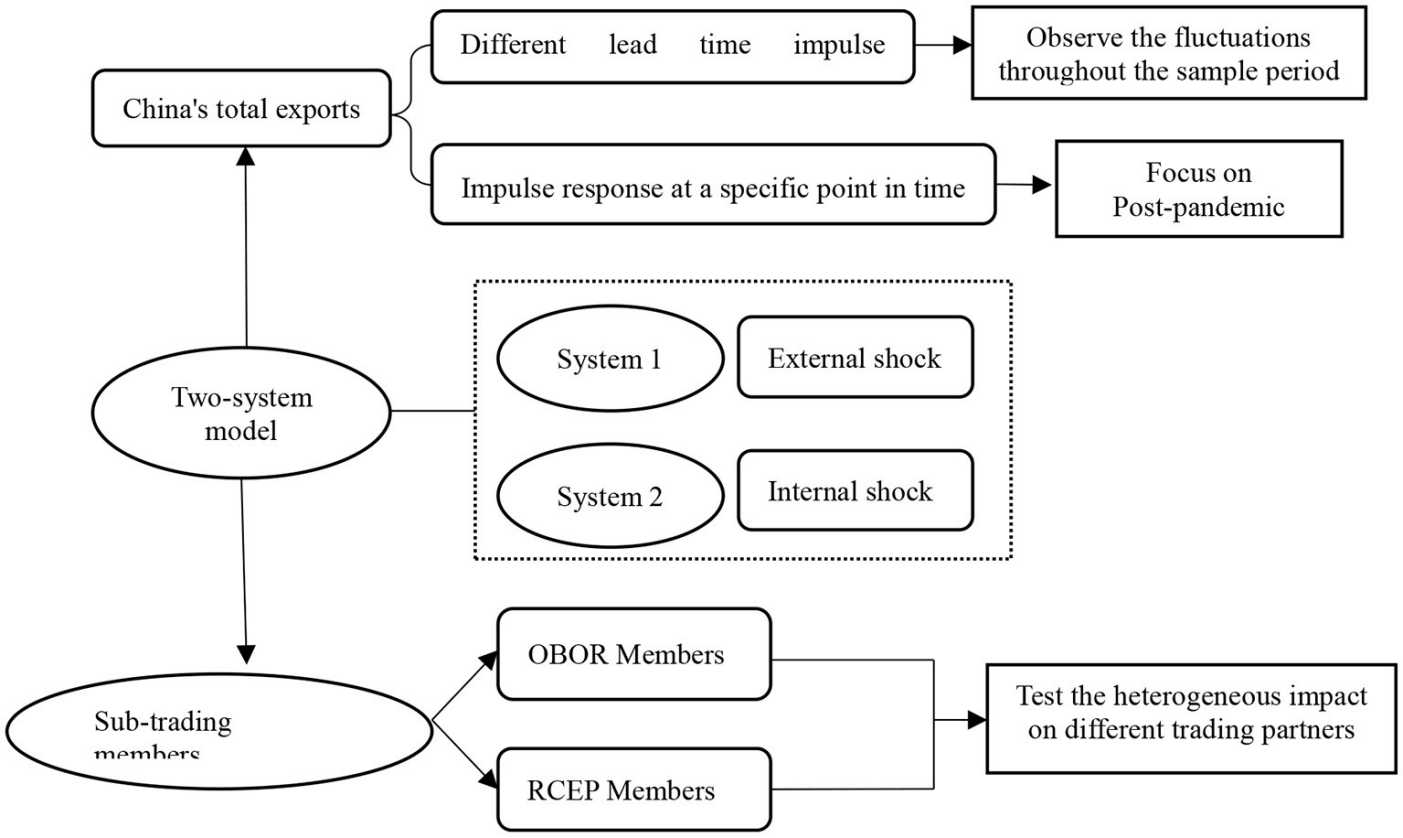
Mind map of the research.

EPU can be global EPU or China's EPU. Global EPU is worked out as the current GDP-weighted mean of the EPU indices of 21 countries and regions, including the U.S., Japan, and China, based on PPP adjustment, with January 1997 as the base period. According to Baker et al. (23), China's EPU, with January 1995 as the base period, is expressed in terms of standard *South China Morning Post* report frequency. Figure 3 provides the mind map of the research.

This paper chooses 232 samples in all from the sample interval from January 2002 to April 2021 in order to better observe the pandemic's shock. Before empirical analysis, we make a seasonal adjustment using the Census X-12-ARIMA method and normalize the parameters in order to eliminate the potential effect of dimensions on model estimation. Table 1 provides the design of variables.

**Table 1:** Variable design and specification.

**Variables**	**Variable description**	**Data source**
Gross exports (exp)	Log of China's actual gross exports	Wind database
Exports to OBOR Members (brex)	Log of China's exports to “One Belt One Road” members	CEInet statistics database
Exports to RCEP Members (pex)	Log of China's exports to RCEP members	CEInet statistics database
Global economic policy uncertainty (gepu)	Global economic policy uncertainty index	http://www.policyuncertainty.com
China economic policy uncertainty (cepu)	China economic policy uncertainty index	http://www.policyuncertainty.com
Industrial output (iav)	Log of the month-on-year added value of industrial enterprises above a designated size	Wind database
RMB exchange rate (rate)	Average exchange rate of RMB to USD	Wind database

## Empirical Findings Analysis

### Stationarity and Co-Integration Analysis

An augmented Dickey-Fuller (ADF) unit root test is conducted prior to parameter estimation. The result in Table 2 shows that except for global EPU, the horizontal series of all other variables are non-stationary. The first-order differential equations of the variables are chosen for a test and it is found that the equations are stationary.

**Table 2:** ADF unit root test result.

	**Variables**	**ADF value**	**5% significance level**	***P*-value**	**Conclusion**
Horizontal series	exp	−2.557933	−3.429570	0.3002	Unstable
	gepu	−4.483984	−3.429398	0.0020	Stable
	cepu	−2.448088	−3.429570	0.3538	Unstable
	iav	−3.283555	−3.430572	0.0717	Unstable
	rate	−0.924452	−3.429484	0.9504	Unstable
Difference series	exp	−17.36020	−3.429570	0.0000	Stable
	gepu	−18.09880	−3.429484	0.0000	Stable
	cepu	−16.68055	−3.429570	0.0000	Stable
	iav	−6.064684	−3.430669	0.0000	Stable
	rate	−10.66402	−3.429484	0.0000	Stable

Considering the use of differential equations of the variables may cause the loss of a large amount of data and the leak of key information, the co-integration analysis of the variables is once again conducted. Even if they are co-integrated, i.e., the horizontal series are non-stationary, the TVP-SV-VAR model still applies. The result of the Johansen co-integration test in Table 3 shows that because each variable has at least one co-integration relationship, their horizontal series can be chosen for estimation.

**Table 3:** Johansen co-integration test result.

	**Null hypothesis**	**Characteristic value**	**Trace statistic**	**5% significance level**	***P*-value**
	None	0.142537	65.49697	63.87610	0.0363
	At most 1	0.070957	30.58951	42.91525	0.4676
System 1	At most 2	0.040383	13.88229	25.87211	0.6673
	At most 3	0.019737	4.525035	12.51798	0.6654
	None	0.134183	64.08419	63.87610	0.0480
System 2	At most 1	0.076691	31.37769	42.91525	0.4226
	At most 2	0.037273	13.26502	25.87211	0.7173
	At most 3	0.020244	4.62436	12.51798	0.6482

*System 1 variables include: gepu, exp, iav, and rate*.

*System 2 variables include: cepu, exp, iav, and rate*.

### Parameter Estimation Sample Analysis

A TVP-SV-VAR model with a second-order lag is built. MCMC is used to make 10,000 samplings and the first 1,000 burn-ins are rejected so as to prevent non-stationarity of early iterations in effective sampling. As is shown by the parameter estimation result in Table 4, each parameter has a mean value located in a 95% confidence interval and the Geweke value is smaller than 1.96, indicating that the null hypothesis that the parameters converging to the a posteriori distribution cannot be rejected at a 5% significance level. As for invalid factors, except for sh2 in system 2 which is greater than 100, all others meet the requirement of the a posteriori extrapolation. Overall, MCMC simulation is valid.

**Table 4:** TVP-SV-VAR parameter estimates.

**Epu**	**Parameter**	**Mean**	**Sd**	**95% confidence interval**	**Geweke value**	**Invalid factor**
System 1	sb1	0.0224	0.0026	(0.0181, 0.0281)	0.716	11.36
	sb2	0.0221	0.0025	(0.0178, 0.0276)	0.100	12.04
	sa1	0.0670	0.0327	(0.0362, 0.1556)	0.166	36.71
	sa2	0.0641	0.0347	(0.0325, 0.1519)	0.198	16.58
	sh1	0.5679	0.1083	(0.3881, 0.8027)	0.531	82.23
	sh2	1.6156	0.2040	(1.2357, 2.0314)	0.368	82.44
System 2	sb1	0.0228	0.0027	(0.0183, 0.0285)	0.686	15.22
	sb2	0.0218	0.0023	(0.0178, 0.0268)	0.482	10.72
	sa1	0.0767	00334	(0.0397, 0.1612)	0.047	35.82
	sa2	0.0583	0.0276	(0.0312, 0.1296)	0.964	26.89
	sh1	0.2960	0.0781	(0.2100, 0.4850)	0.000	96.46
	sh2	1.6503	0.2275	(1.2456, 2.1230)	0.763	126.23

*The settings of system 1 variables and system 2 variables are the same as Table 3*.

### Impulse Response Analysis

#### Gross Export's Shock on EPU

**Figure 4:**
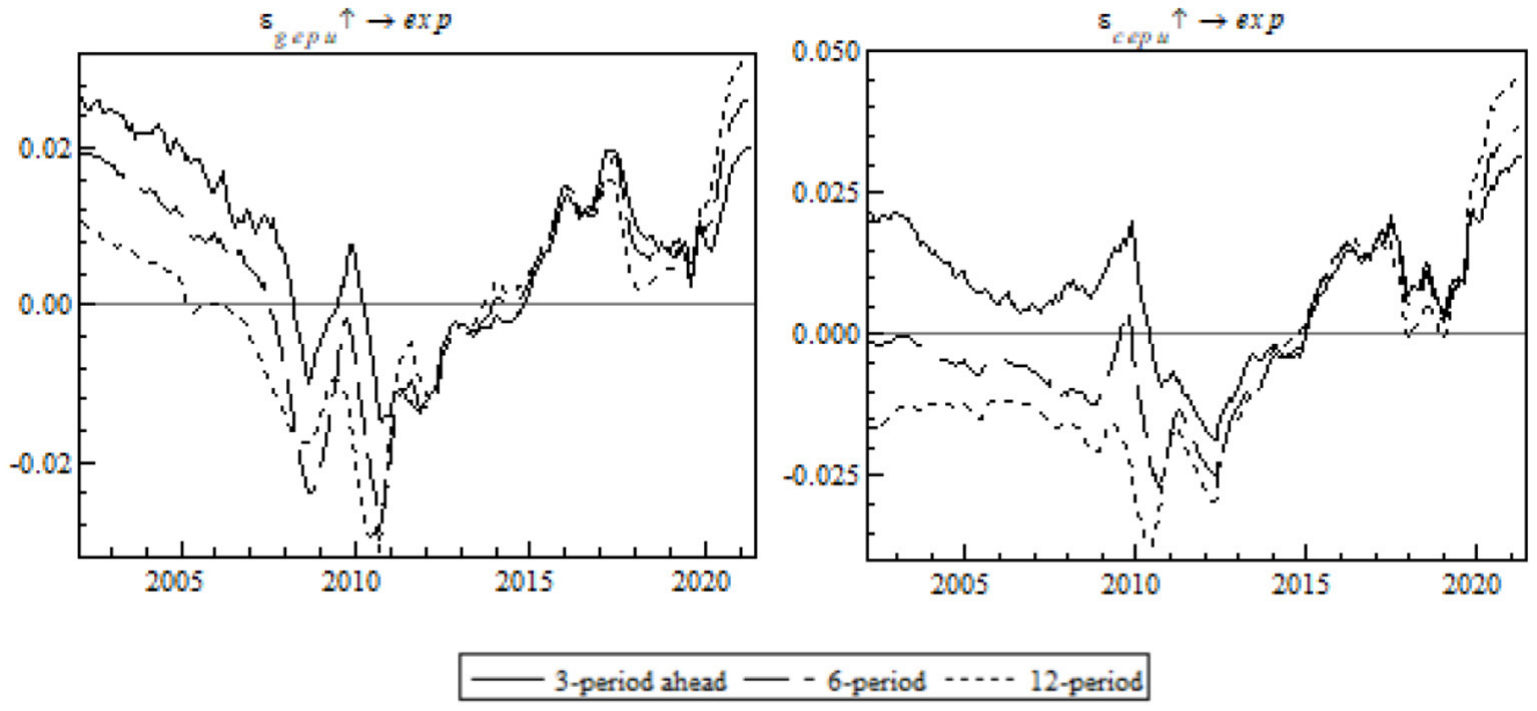
Equal-interval impulse response of China's gross exports to EPU.

As shown in Figure 4, the following provides impulse response analyses with different periods. For the model, there are three periods set up, i.e., short-term (3-month), mid-term (6-month), and long-term (12-month), in order to observe the dynamic time-varying characteristics of the shock on China's gross exports.

On the whole, the impulse response curve of the shock of EPU on China's gross exports is obviously trended and fluctuating, especially in terms of time-varying characteristics. Seen from the left chart, the impulse response of the shock of global EPU on China's gross exports is greater in the mid-term, and seen from the right chart, the impulse response of the shock of China's EPU on its gross exports in the short-term. The impulse response exhibits structural fluctuations with time. The range of the shock of global EPU on China's gross exports was greater. Before 2005, the shock of global EPU and China's EPU on China's gross exports was stable. In 2005-2008, the impulse response fell by a large margin. From the outbreak of the pandemic in early 2020 to the post-pandemic era, there was an upsurge in EPU after a short-term fall, because the multiple policies taken by the governments against the pandemic had a superimposed effect that contributed directly to a surge in global EPU and China's EPU and drove down China's gross exports at both the supply side and the demand side. Still, the gradual drop in the EPU index resulted in an upsurge in China's gross exports.

**Figure 5:**
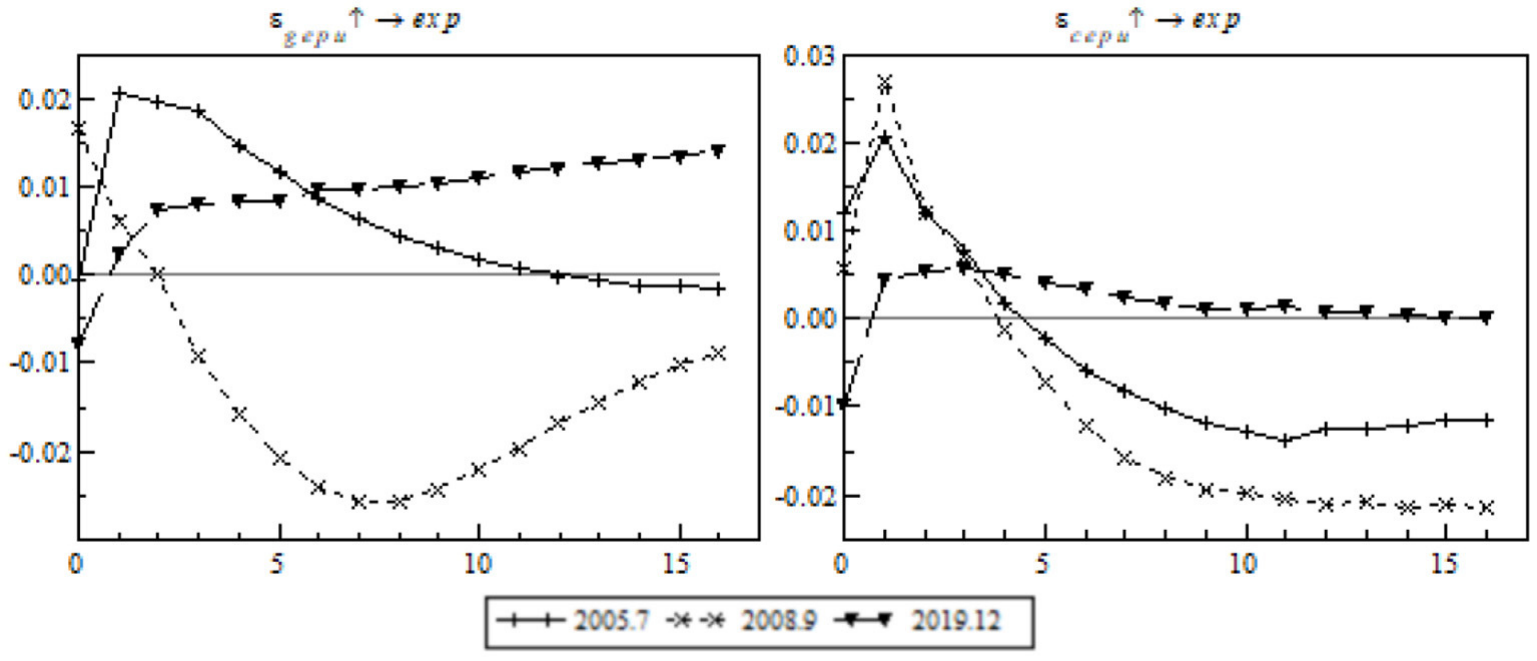
Impulse response of China's gross exports to time point-based EPU.

We will conduct time point-based impulse response analysis. The pandemic is taken as an important event. We can make a comparative analysis between the RMB exchange rate reform and the financial crisis to observe the shock on China's export trade in the post-pandemic era, as shown in Figure 5.

On the whole, the shock response curves of different events and points in time were isolated, indicating that EPU's shock on China's gross exports was remarkably different from those of other events. In the framework of the RMB exchange rate reform, the short-term positive shock of China's gross exports to global EPU was strongest, but it weakened significantly as the lag lengthened, as shown in the left chart; by contrast, the shock of China's EPU was negative all the time after four lags. In the framework of the financial crisis, the negative shock of China's EPU on gross exports to EPU lasted by as many as 12 lags (or for 1 year), indicating that the crisis had a long-term negative response on China's gross exports through the medium of EPU. However, a positive response occurred from the first lag onwards, and the positive response to EPU was reinforced as the lag increased. The phenomenon arose to a great degree from China's effective pandemic prevention measures that contributed to a rapid return to work. The recovery of the upstream supply chain effectively addressed supply inadequacy at the early stage of the pandemic. Global EUP caused the relative shift of global orders to China and ensured China's export trade resumed growth after a short-term shock.

#### Heterogeneity of the Impulse Response Associated With Export Trade to Trade Members

As shown in the above analysis, as opposed to the long-term nature of the shock of EPU on China's export trade in the financial crisis, the shock of China's exports to EPU is of a short-term nature. Here we take the example of the OBOR/RCEP members to investigate the heterogeneity of the shock of global EPU and China's EPU on China's export. For the purpose of comparability, the lag-based equal interval and the specified points in time are the same as the above.

##### OBOR Members

**Figure 6:**
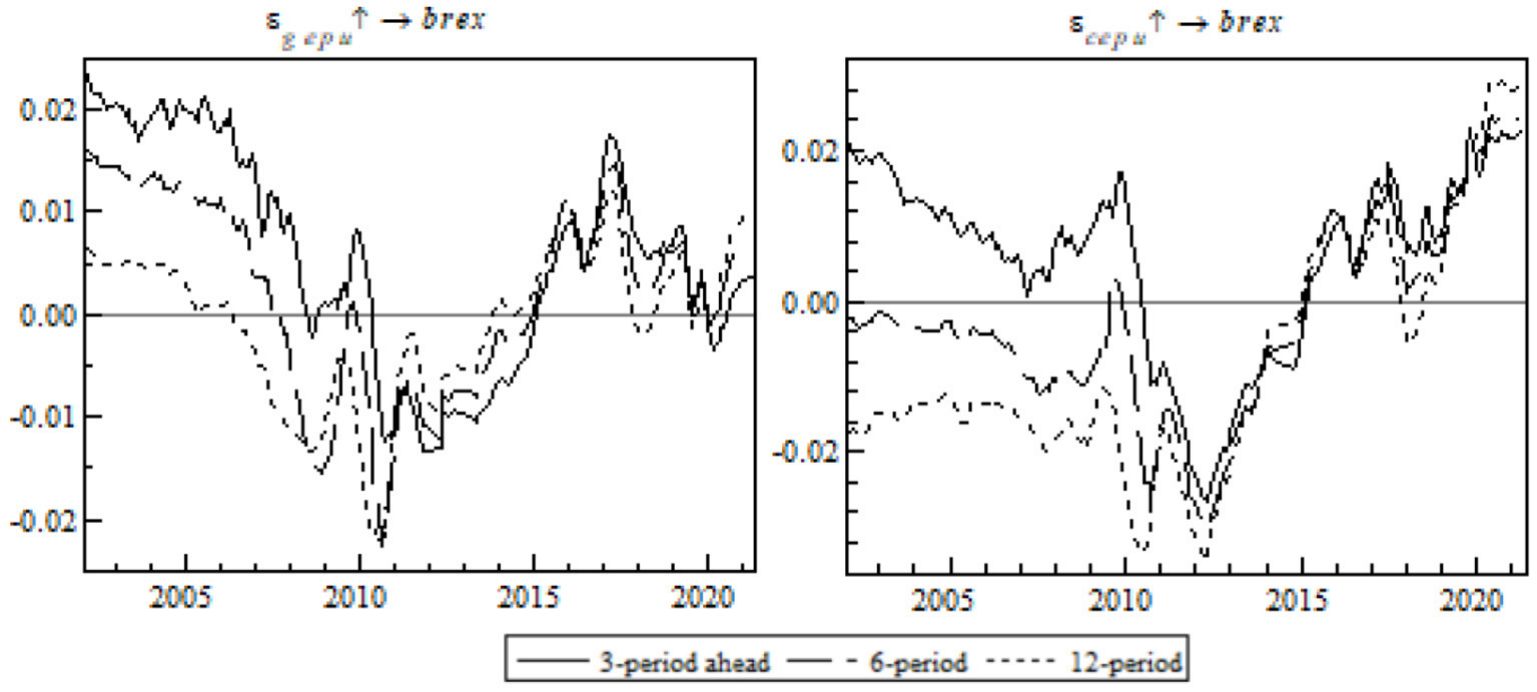
Equal-interval impulse response of export to OBOR members to EPU at equal intervals.

This paper provides an impulse response analysis of different periods. In order to investigate the time-varying characteristics of EPU's shock on China's export to OBOR members, the following observation in Figure 6 provides the trend of the impulse response curve at equal intervals.

From the perspective of the whole sample interval, global EPU and China's EPU had a significant short-term shock on the OBOR members. As the trends show, EPU at early sampling led to gross exports to OBOR changing in the same direction; however, from 2005 onwards, EPU changed in an increasingly intensified reverse direction until 2013 when it resumed stability and climbed in the direction of the zero axis before a negative-to-positive structural change. This can be attributed to the introduction of the 2013 OBOR initiative that intensified China's further communication with the members and reduced the negative shock of EPU on export to the OBOR members.

**Figure 7:**
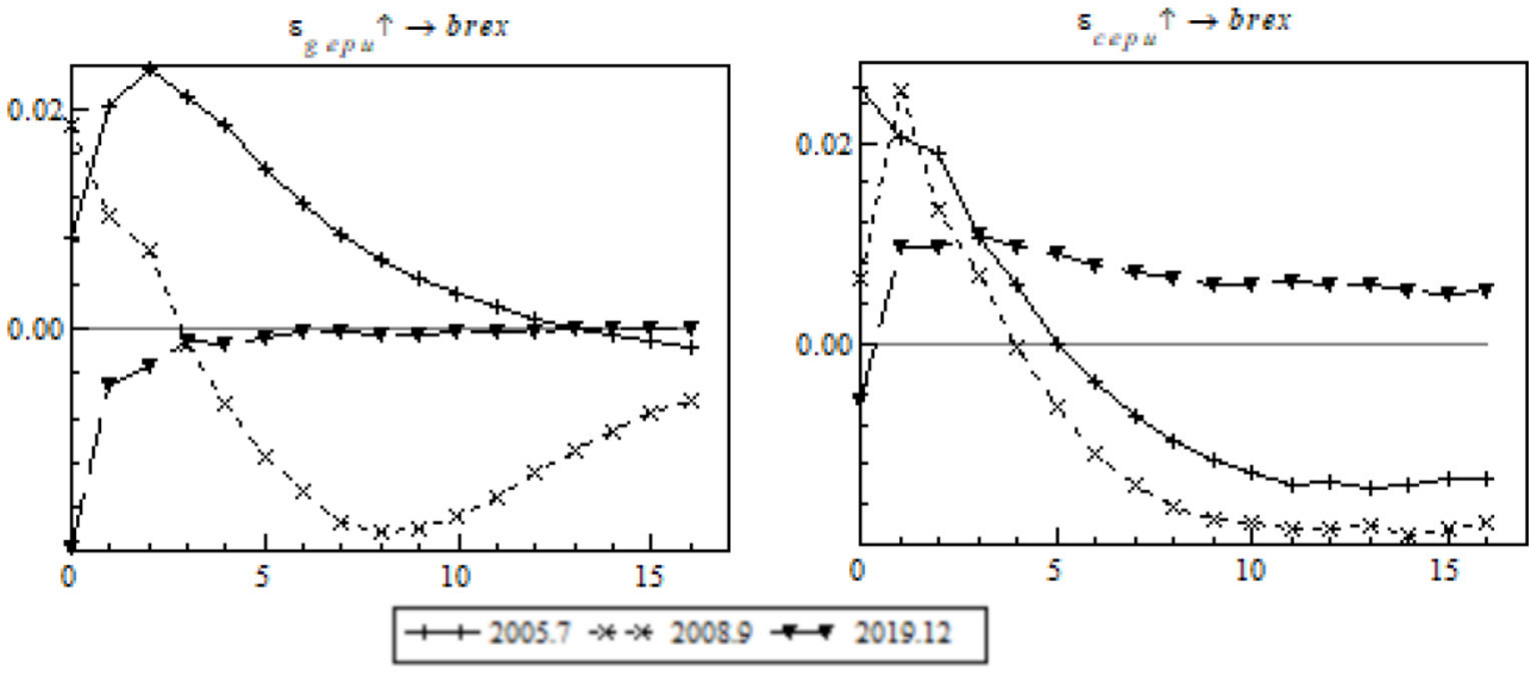
Impulse response of export to OBOR members to time point-based EPU.

Then we will conduct an analysis to analyze EPU's shock at specific points in time. In order to capture the shock of EPU in the post-pandemic era precisely, we focus on the impulse response of export to OBOR members to the pandemic (see Figure 7).

During the pandemic, the shocks of global EPU and China's EPU on export to the OBOR members differ. The negative impulse response of export to global EPU is greater than China's EPU (see the left chart). All negative impulse responses occurred as short-term responses and those of the contrast events were primarily mid-term or long-term in nature. It is probably because the pandemic negatively affected the global trade environment, led to damage to the global supply chain and industries, and dealt a heavy short-term blow to China's export to its OBOR members. Fortunately, the OBOR policy buffered the adverse shock arising from the pandemic, the trade facilitation-associated dividend defended against multiple external risks, and China took advantage of the regional cooperation platform to propel economic policy coordination among the OBOR members. Besides, the dawning of the post-pandemic era is expected to still the export fluctuations quickly.

##### RCEP Members

**Figure 8:**
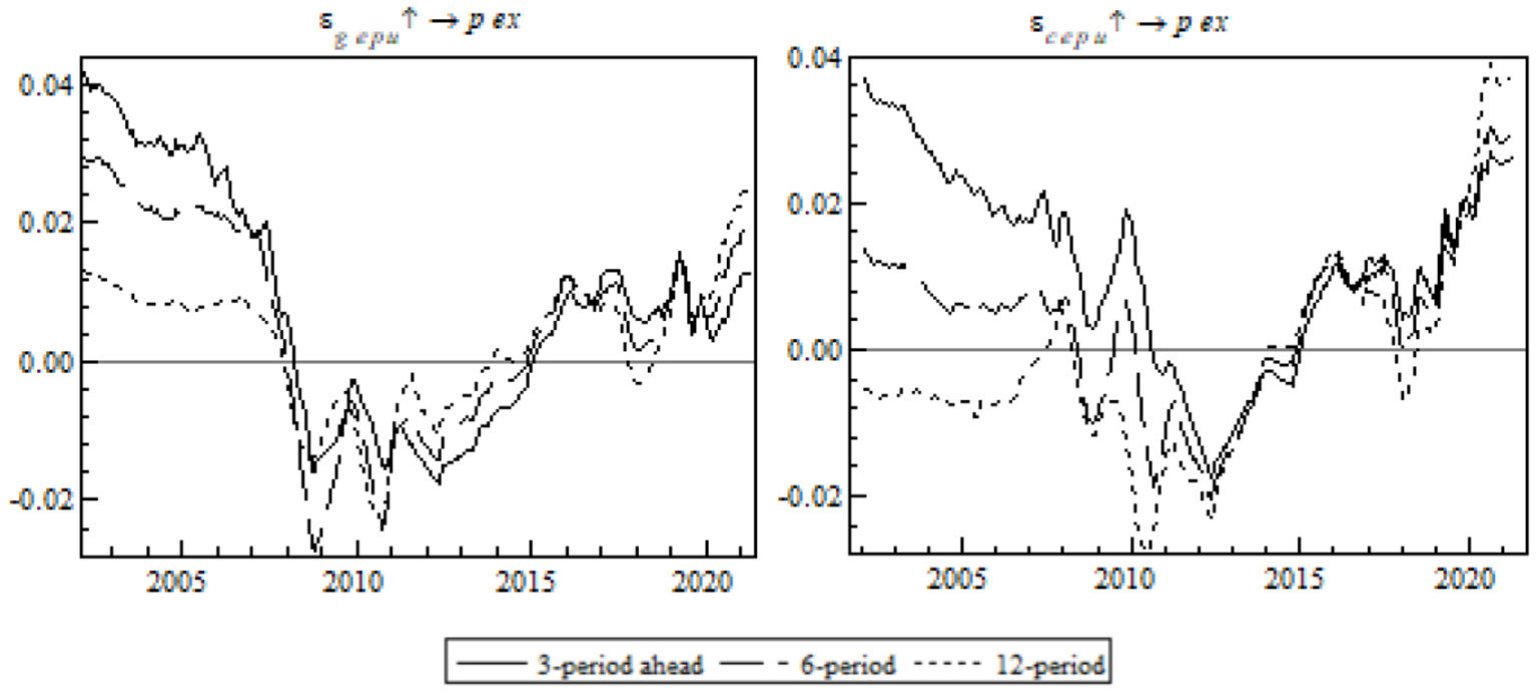
Equal-interval impulse response of China's export to RCEP members to EPU.

The following is an impulse response analysis based on different periods. A unit of positive shock was exerted on each of the three periods at each time point to observe the impulse response of China's export to the RCEP members at each time point (see Figure 8).

In the temporal dimension, the shock of global EPU on China's export to the RCEP members proves strongest in the mid-term (see the left chart) and the shock of China's EPU proves strongest in the short-term (see the right chart). The shock of EPU on China's export to the RCEP members took a remarkable turn in 2012, very likely because of a free trade agreement signed by ASEAN, China, and Japan the same year. The agreement provided external propulsion to the formation of RCEP, therefore sending a positive signal to other regional members to smooth the negative effect of EPU and increase China's gross exports to the RCEP members. However, the pandemic brought about a short-term fall in the impulse response in both systems, and the fall was especially big because of the shock of global EPU.

**Figure 9:**
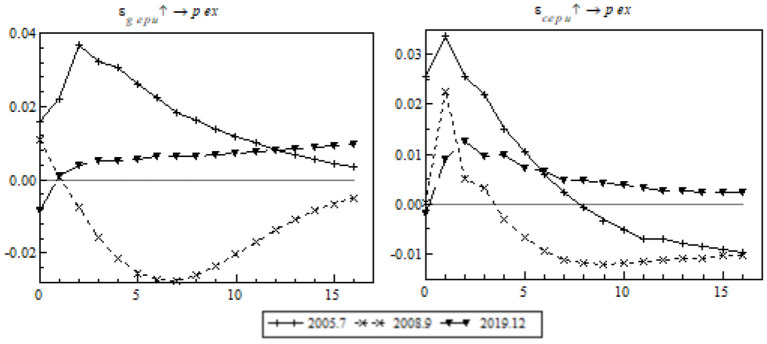
Time point-based impulse response of export to RCEP members to EPU.

We will carry out an impulse response analysis at specific time points. A standard unit of positive shock was exerted on EPU in order to observe China's gross exports to the RCEP members as they changed with time in the post-pandemic era (see Figure 9).

Unlike the contrast event where the mid- and long-term structural shock switched from positive to negative, EPU's shock on export to the RCEP members changed quickly from negative to positive in the short-term. This is in line with the expectations. Apart from effective preventive measures, China coordinated the initiation of RCEP and provided a more stable business-friendly environment. The transparent mechanism in the free trade agreement helped China circumvent EPU-associated export risks, consolidate the export supply chain, and restore its trade relations with other RCEP members step by step.

### Robustness Test

#### Replacement of the Method of Measurement of the EPU Index

**Figure 10:**
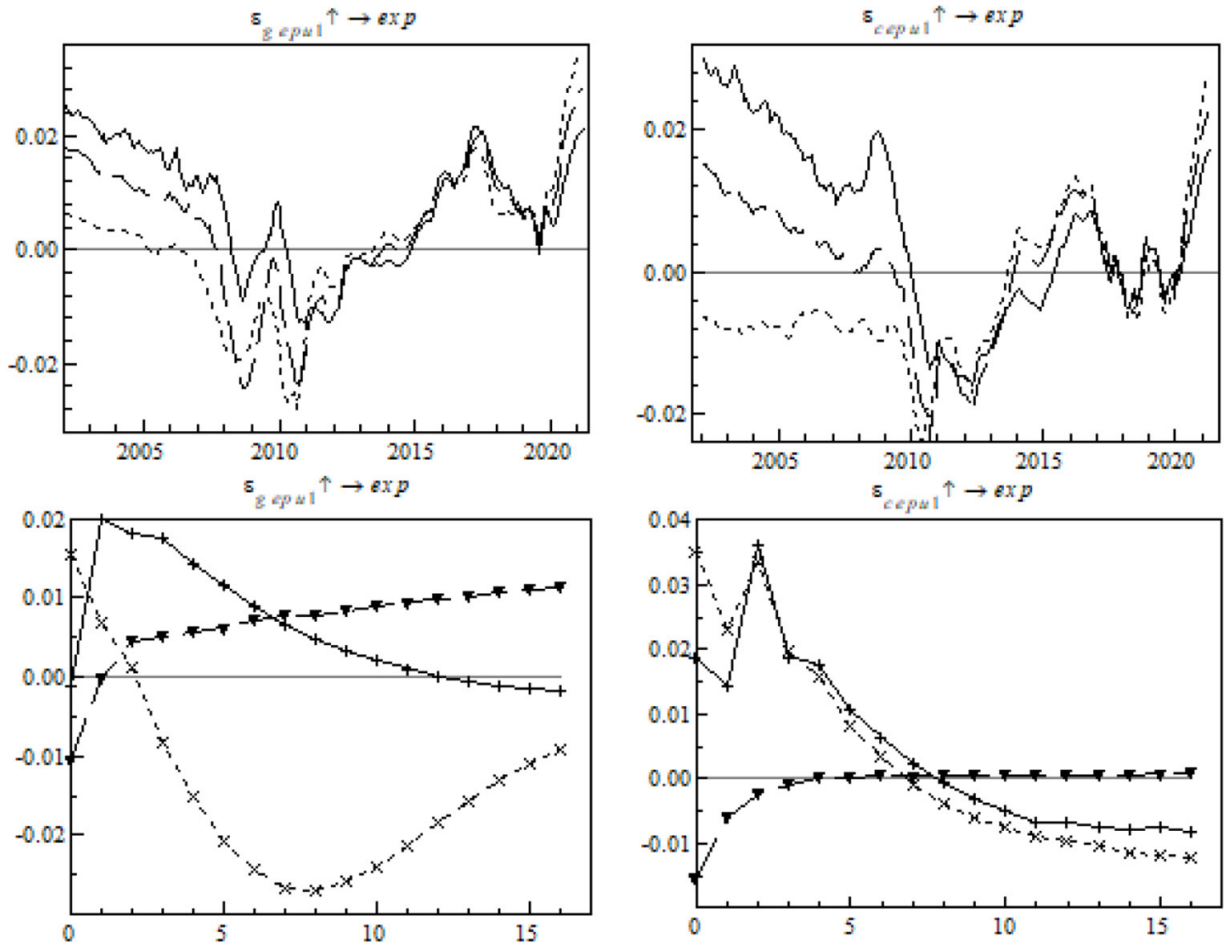
Impulse response of China's gross exports to the change in the EPU measurement index.

In order to eliminate the measurement deviations of the EPU index, this paper redefines two indices: the global EPU index is replaced with GDP at current prices, and China's EPU index is replaced with the quantitative index based on Chinese mainland newspapers *Guangming Daily* and *People's Daily* (24). As shown in Figure 10, there was basically no change in the fluctuation trend and shock direction of global EPU and China's EPU. In this respect, the replaced index was the same as the pre-replacement index. China's gross exports remained the most remarkable from the short-term negative shock from EPU, so the model passed the robustness test satisfactorily.

#### Adjustment of the Order of Variables in the Model

**Figure 11:**
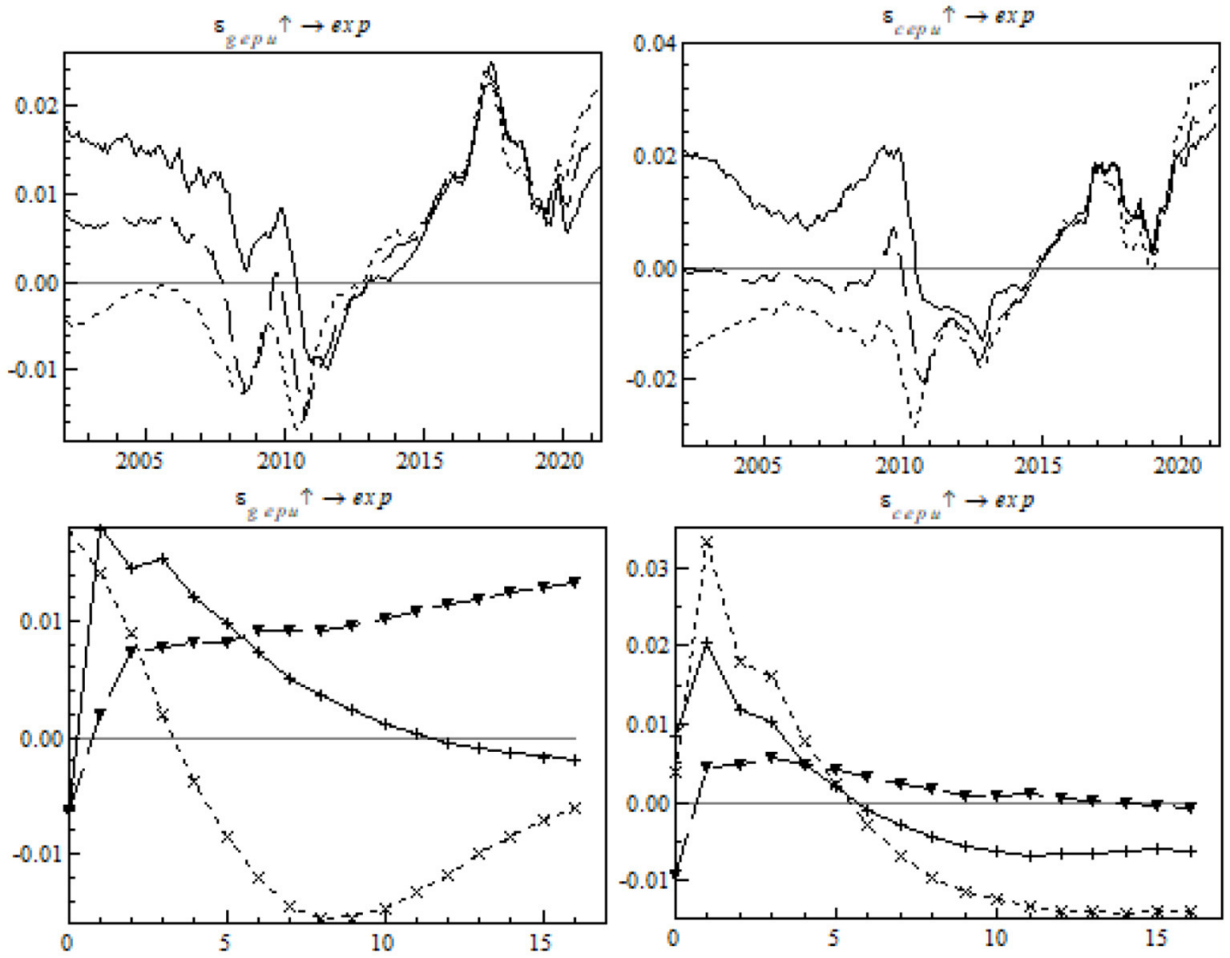
Impulse response of China's gross exports to EPU after variable readjustment.

We can extrapolate from the TVP-SV-VAR model that the order of the endogenous variables in the model will affect the impulse response. In order to investigate the impact, gross output, the RMB exchange rate, global EPU, China's EPU, and China's gross exports were rearranged for parameter estimation. As shown in Figure 11, there was no obvious difference in impulse response from the original findings before the readjustment. It indicates that the aforesaid core conclusion is tenable.

## Conclusion and Enlightenment

This paper uses the continuous monthly data from January 2002 through April 2021 to build a TVP-SV-VAR model and apply it to two systems. The pandemic is taken as an important event, and the RMB exchange rate reform and the financial crisis are taken as contrast events to study the shock of China's EPU on China's gross exports (including exports to the OBOR/RCEP members) in the post-pandemic era. The empirical analysis returns three conclusions. First, on the whole, the shock of EPU on China's gross exports is time-varying, different, and is remarkably structural. Second, from the perspective of the whole sample interval, EPU has a significant short- to mid-term shock on China's gross exports and export to the OBOR/RCEP members. Third, in the post-pandemic era, the short-term shock on China's gross exports and export to OBOR/RCEP members is subject mainly to EPU, especially the negative shock of global EPU.

At the critical juncture of post-pandemic economic revival, China should take action more actively to respond to the dual-circular challenge and the increasingly complicated international economic situation. That is why it is important for China to study EPU's impact on export and find appropriate solutions. First, free trade should be promoted and international bilateral/multilateral trade dialogues should be introduced so that China can benefit from a stable and well-regulated trade environment. Second, both external and internal economic risks should be more closely monitored and controlled, and sustainable economic policies should be made for the pandemic in order to ensure the export market develops stably and in an orderly way. Third, China should continue to push ahead with the OBOR initiative, apply RCEP agreement stipulations, and employ regional advantages to eliminate EPU-associated export risks.

## Data Availability Statement

The original contributions presented in the study are included in the article/supplementary material, further inquiries can be directed to the corresponding author.

## Author Contributions

GH: conceptualization, methodology, formal analysis, writing—original draft preparation, project management, and funding acquisition. SL: conceptualization, methodology, writing—original draft preparation, and funding acquisition. All authors have read and agreed to the published version of the manuscript.

## Funding

The authors acknowledge financial supports from National Social Science Fund of China (award number 21BJY084).

## Conflict of Interest

The authors declare that the research was conducted in the absence of any commercial or financial relationships that could be construed as a potential conflict of interest.

## Publisher's Note

All claims expressed in this article are solely those of the authors and do not necessarily represent those of their affiliated organizations, or those of the publisher, the editors and the reviewers. Any product that may be evaluated in this article, or claim that may be made by its manufacturer, is not guaranteed or endorsed by the publisher.
